# Lessons identified from initiating a thalassaemia programme in a conflict setting: a case study from northeast Syria

**DOI:** 10.1186/s13031-023-00503-2

**Published:** 2023-02-07

**Authors:** Sally MacVinish, Crystal van Leeuwen, Maartje Hoetjes, Yoshihiro Aoki, Deirdre Foley, Harriet Roggeveen

**Affiliations:** 1 Médecins Sans Frontières, Amsterdam, The Netherlands; 2 School of Tropical Medicine and Global Health, Nagasaki University, Nagasaki, Japan; 3 Department of Paediatric Infectious Disease, Children’s Health Ireland at Crumlin, Dublin, Ireland

**Keywords:** Thalassaemia, Syria, Conflict, Medical programming, Iron chelation therapy, Blood transfusion, Humanitarian

## Abstract

**Background:**

Thalassaemia affects many families in Northeast Syria, an area devastated by over a decade of conflict which has significantly impacted their health system. People with thalassaemia require holistic multidisciplinary care for the clinical complications of thalassaemia. The risks of thalassaemia treatment include blood-borne viral infections secondary to unsafe transfusion, increased vulnerability to serious bacterial infection following splenectomy, and complications of both iron overload and iron chelation therapy. Médecins Sans Frontières (MSF) provided outpatient thalassaemia care programmes in northeast Syria between April 2017 October 2019 in a complex conflict context challenged by population displacement, the destruction of medical facilities, and periods of insecurity.

**Methods:**

We performed a secondary descriptive analysis of the thalassaemia cohort data to describe basic clinical and demographic characteristics of the patient population. A desk review of internal and publicly available documents was supplemented by informal interviews with MSF staff to describe and analyse the programmatic approach.

**Case description:**

MSF delivered programmes with thalassaemia investigations, provision of blood transfusion, iron chelation therapy, and psychosocial support. Thalassemia programmes were novel for the organisation and operational learning took place alongside service implementation. Lessons were identified on equipment procurement and the requirements for the implementation of vital investigations (including ferritin testing), to inform clinical decision making. Lessons included the importance of supply planning for sufficient blood products to meet diverse clinical needs in a conflict area, so those with thalassaemia have continued access to blood products among the competing priorities. Iron chelation therapy met a large need in this cohort. Adapted protocols were implemented to balance social factors, hygiene considerations, toxicity, tolerability, and adherence to therapy. Wider service needs included considerations for family planning advice and services, continuity of care and patient access through decentralised services or laboratory access, psychosocial support, and improved data collection including quality of life measurements to understand the full impact of such programmes.

**Conclusions:**

Although this type of programming was not “routine” for the organisation, MSF demonstrated that life-sustaining thalassaemia care can be provided in complex conflict settings. International non-governmental organisations can consider this care possible in similar contexts.

## Background

Thalassaemia affects many families in Northeast Syria (NES), an area devasted by over a decade of conflict which has significantly impacted their health system [1, 2]. It is reported that 5% of people in Syria are carriers of the β-thalassaemia gene and between 1 and 5% are carriers of the α-thalassaemia gene [3]. People with thalassaemia require holistic multidisciplinary care for the early and late clinical complications of thalassaemia; including chronic anaemia, hepatosplenomegaly, and extra-medullary haematopoiesis. In addition, we must consider the side effects of thalassaemia treatment, which include blood-borne viral infections secondary to transfusion, increased vulnerability to serious bacterial infection following splenectomy, and complications of both iron overload and iron chelation therapy (ICT) [1, 4]. In resource-limited settings, with inadequate access to blood transfusion and ICT, less than half of thalassaemia patients live to age 30 [4].

Given the multisystem nature of complications from thalassaemia and its treatment, international guidelines in place at the time of programming recommended the following for the organisation and programming of thalassaemia care:

1. Day care services with access to inpatient facilities.
2. Facilitation of equal access to quality care for every thalassaemia patient.
3. Close collaboration with other services, such as the blood bank and laboratories.
4. Evidence-based guidelines or standards, providing comprehensive and holistic care.
5. Active involvement in research programmes.
6. Collaboration with patient support groups.
7. Advocacy to health authorities for service development and patients’ rights [5].

As an external medical humanitarian emergency organisation, Médecins Sans Frontières (MSF) has the additional programming element of collaboration with local staff, communities and health actors, which is not considered in international guidelines. The challenges, necessity, and positive impact on programme effectiveness, and respect for the community and their dignity of working with local actors in different settings has been discussed by MSF authors in the literature [6].

MSF ran thalassaemia programmes in NES from April 2017 during a period of large-scale population displacement and health system disruption. Prior to this, from November 2016, the Syrian Democratic Forces (SDF), with support from the United States of America, seized control over large parts of Raqqa governorate from the Islamic State [7]. During this period the Raqqa governate had over 50,000 people internally displaced, of which 20,000 were displaced in March of 2017 [8]. It is reported that 322,100 displacements from and within Raqqa governorate were recorded in 2017. In October 2017 SDF forces gained control over Raqqa city and the previously displaced population began to return in the following months [9]. Following this, there was a relatively stable period in the areas MSF was working, until the start of a military offensive by Government of Turkey in October 2019 which displaced more than 190,000 people and ended MSF’s thalassaemia activities [10].

As the conflict in Syria has become protracted, international organisations have diversified activities from emergency care to include care of chronic diseases. MSF patients and healthcare staff reported that, prior to the conflict, Syria’s national thalassaemia programme provided free pre-natal counselling, safe blood transfusion, and ICT [1]. Between April 2017 and October 2019 when MSF was running thalassaemia programmes, most of the pre-existing thalassaemia treatment centres in NES were closed due to a lack of equipment, medical supply, and staffing, and likely impacted by the insecurity affecting the wider health system [1, 4]. Access to healthcare for some was further disrupted by displacement, with some families displaced on multiple occasions [1, 11]. In addition there were challenges for some to move into Government of Syria controlled areas. The need for thalassaemia care was clear. Data from MSF activities in Tal Abyad (also known as Girê Spi) and Kobanê (also known as Ayn al-Arab) show that thalassaemia was recorded in 0.5% of the under 5 years out-patient consultations in 2014, and 3.4% of the 2013–2014 paediatric in-patient admissions (patients under 18 years), although a comprehensive thalassaemia programme was not provided by MSF during this time [12].

After requests from the community and in-depth programming discussion, MSF engaged in thalassaemia activities in two NES locations—Menbij city and Tal Abyad city. Outpatient thalassaemia programmes ran in Menbij between April 2017 and April 2018 and in Tal Abyad between October 2017 and October 2019. Holistic care was provided, including splenectomy and ICT. ICT was provided with the aim of minimising some iron overload complications, acting as a ‘bridge’ until the health authorities could restart activities. However, in October 2019, military operations led to renewed fighting and insecurity [13]. The MSF Tal Abyad project was subsequently closed as access was no longer possible and MSF-provided thalassaemia care ceased. Since then, MSF has continued to work alongside other health actors in NES, although is no longer providing thalassaemia care. MSF runs a thalassaemia programme in Lebanon for Syrian refugees, including those who have fled from NES [14].

Evidence on the most effective way of delivering programmes for thalassaemia in humanitarian settings is limited. A 2015 evidence review of health interventions in humanitarian settings highlighted the need for more evidence on how to implement or deliver non-communicable disease programmes in these contexts, especially around continuity of care [15]. While there is evidence describing splenectomy treatment of children with thalassaemia in an Afghanistan military hospital [16], evidence to guide holistic thalassaemia programming, including the initiation of the provision of ICT, in conflict settings is lacking. To the best of our knowledge, MSF was the only non-governmental organisation providing comprehensive thalassaemia care, including the provision of ICT, in the non-Syrian government-controlled areas of NES between April 2017 and October 2019. The aim of this case study is to share key lessons identified from the implementation of MSF’s thalassaemia programming approaches in this context and time period. The objectives are to:

1. Describe the thalassaemia patient cohort.
2. Describe MSF’s programming approach.
3. Analyse the challenges and enablers to successful programming, and share reflections, lessons identified, and recommendations for thalassaemia programmes in conflict settings.

## Methods

A secondary analysis of clinical MSF programme data was conducted to describe demographic and simple clinical characteristics of the thalassaemia patient cohort. A desk review of internal MSF documents was performed including review of project proposals, monthly medical reports, bi-annual planning documents, and ad hoc reports on specific aspects of the programme, for example ICT and data management. In addition, journal publications describing MSF activities and MSF media communications on NES were reviewed. From these sources, authors generated a descriptive timeline and identified the key aspects of thalassaemia programming, relevant contextual events, and other challenges and enablers to programme implementation. Telephone and video discussions were held with eight MSF staff who worked on NES thalassaemia programmes in project level provision of clinical care, country level health programming, and headquarters management and advisory positions. Their views were sought on key programming themes, strengths, weaknesses, recommendations and the interview notes supplemented information from the secondary data analysis and desk review. Interviews with staff were conducted to supplement other findings for an MSF programmatic review, as opposed to undertaking key informant interviews and analysis. Author CvL, previous country level health programming coordinator and subsequant headquarters responsible for MSF’s health activities in NES, provided guidance on topics of interest for programmatic lessons and supplemented findings from other data sources. Due to MSF’s rapid exit from the area it was not possible to include patients, families, and local Syrian staff in interviews, discussions, or writing of this case study.

The quantitative component of this case study fulfilled the exemption criteria set by the MSF Ethics Review Board (ERB) for a posteriori analysis of routinely collected clinical data, and thus did not require MSF ERB review. MSF ERB review was waived for the qualitative interviews with MSF staff by the Medical Director. However, verbal consent was obtained prior to telephone and video discussions with MSF staff and written consent was retrospectively obtained from staff to include their views in an external publication.

## Case description

### MSF thalassaemia cohort basic clinical and demographic characteristics

As of September 2019, data were available for 378 thalassaemia patients registered in the MSF Tal Abyad clinic programme between 2017 and 2019. This included 10.3% (n = 39) patients recorded as previously registered at the MSF-supported Menbij thalassaemia clinic. Clinical and demographic characteristics of the cohort are described in Table 1. Of the 333 patients who had a residency status recorded, 75% (n = 251) were living in their area of origin and 25% (n = 82) identified as internally displaced persons.

**Table 1:** MSF thalassaemia patient cohort recorded demographic and clinical characteristics September 2019

Sex	Female 50.3% (n = 190)							
Clinical status	Active* 74.3% (n = 281)			Non-active* 23.8% (n = 90)		Died 1.9% (n = 7)		
Age	< 1 year 2.4% (n = 9)	1–5 years 28.6% (n = 108)		6–10 years 31% (n = 117)	11–15 years 20.4% (n = 77)	16–20 years 11.1% (n = 42)	> 20 years 6.6% (n = 25)	
							Range: 0.3–39 years	
							Median: 9.7 years	
Governorate	Raqqa 70.1% (n = 265)		Aleppo 25.7% (n = 97)		Deir es-Zor and Al Hassekah^ 2.4% (n = 9)		Unidentifiable 1.9% (n = 7)	

*Patients were categorised as ‘active’ if they had attended the thalassaemia clinic within the past five months, and ‘non-active’ if they last attended more than five months ago. ^Governorates combined to suppress small numbers

### Thalassaemia care in in MSF’s programmes in Northeast Syria

#### Clinical investigations

A definitive diagnosis of thalassaemia was not possible as haemoglobin electrophoresis was not available. Patients were usually referred or presented with a prior diagnosis. MSF clinicians confirmed thalassaemia clinically using personal and family history, blood tests (haemoglobin and ferritin), and physical examination for signs of hepatosplenomegaly, congestive cardiac failure, delayed growth and puberty, and facial bone deformity. Other causes of anaemia were sometimes difficult to distinguish clinically, and high rates of iron deficiency were evident secondary to food supply disruption and undernutrition. The distinction between thalassaemia and other causes of anaemia was particularly challenging in children under three months of age. For those who were fed formula milk, the anaemia may be partly explained by caregivers resorting to give over-diluted feeds due to a lack of supply. Therefore, some patients within the cohort were misdiagnosed on initial presentation. Staff reflected that better availability of blood films and training on their interpretation could have aided diagnostic decision-making.

MSF supported laboratory services with supplies, staff, supervision, and training, which led to improvements in practice over time. Tests available relevant to thalassaemia care included point-of-care haemoglobin, full blood count with differentiated white blood cells, ferritin, liver and renal function tests, biochemistry, thyroid stimulating hormone, HbA1c, human immunodeficiency virus (HIV), HCV and hepatitis B virus, syphilis, bedside confirmation of corresponding donor and recipient blood types, and laboratory ABO blood typing and rhesus antigen.

Magnetic resonance imaging which can be used for more accurate measurement of cardiac and liver iron overload was not available [4]. Instead, and acknowledging its limitations, serum ferritin levels were used to estimate iron overload and monitor treatment in routine thalassaemia care. However, as provision of ferritin testing was novel for MSF, approved suppliers were not internally available when programming began. Additionally, the required testing equipment could not be purchased within NES and fragile supply routes into the area were affected by regional political dynamics. Given the inadequate access to ICT, and that patients had received over the threshold number of transfusions (greater than ten units of packed red-cell units [4]), it was expected that ICT would be indicated for all people in the transfusion-dependent thalassaemia cohort. MSF took a pragmatic risk-balanced approach which allowed for the commencement of ICT prior to ferritin testing being available. This was considered appropriate for transfusion-dependent patients that would otherwise receive no ICT, especially with routinely available clinical information which assisted with estimating cardiac overload to inform drug choice. Retrospective pre-chelation ferritin testing on 155 frozen patient serum samples supported this approach, as 87% (n = 136) of patients had a ferritin level above ICT treatment threshold (1000 ng/L) [17]. Before ferritin testing was available, decisions on which ICT medications patients received were based on a risk assessment of their home situation including hygiene considerations and ability to safely use a subcutaneous pump, transfusion frequency, and cardiac functioning. Cardiac overload was assessed based on signs including dyspnoea, peripheral oedema, and hepatomegaly, and investigations including left ventricular hypertrophy on electrocardiogram, and cardiomegaly on chest x-ray. Access to echocardiogram was limited and not routinely available for all patients. Given the importance of ferritin testing for clinical decision-making on ICT, it should be a programming priority to establish the requirements for ferritin-related equipment procurement and transportation, including sanctions-related importation issues early on. Once ferritin testing was implemented, staff used results and clinical criteria to determine which ICT medication was most suitable, although individual patient monitoring of ferritin levels to guide the adjustment of ICT medication and dosage was challenged by clinical data management issues.

Despite MSF support to laboratory services, competing priorities within the hospital led to delays in receiving results for some thalassaemia patients. Some patients with difficult or long travel routes had to leave the thalassaemia clinic to travel home before clinical decision making could be made based on their laboratory results.

Genetic testing was not available in the region. Instead, MSF educated caregivers and patients on the genetic basis of the disease and offered contraception to women. Clinicians occasionally faced questions from patients on family planning but lacked a full service to adequately support them.

#### Safe blood transfusion

MSF improved the quality and safety of transfusion services by supporting blood banks and laboratories with staff, supplies, supervision, training, and standard protocols. Each patient’s frequency of transfusion was determined by their serum haemoglobin levels, aiming for a pre-transfusion haemoglobin of 90–105 g/L by regular transfusion, and 100–120 g/L for those with cardiac problems. MSF capitalised on already well engrained community engagement activities to bolster blood supplies through blood donation awareness activities. Community activities included ‘Thalassaemia Day’ and ‘Blood Donor Day’ celebrations, radio adverts, donor rewards programme, and mobile blood donation drives in multiple locations. Understanding population behaviours and contextual factors was key to anticipating periods of low donation (e.g., Ramadan) and activities, such as blood drives, to increase blood donations beforehand were prioritised. Despite these efforts, blood products were not always in adequate supply. It was particularly difficult to maintain supply when younger men, who were regular donors, left the area to join the conflict. During periods of active conflict, influxes of trauma patients increased demand for blood products. Security risks sometimes limited movements regionally, hindering MSF’s ability to seek donations from areas further away from the hospital. Patients’ families were often a source of blood donation but, as thalassaemia is an inherited condition, some families had more than one person affected and not enough blood to donate to meet all the needs of their family members.

Though packed red-cell (PRC) units are preferable for thalassaemia patients as they are less likely to lead to transfusion reactions [18], and Syrian patients and clinicians are more familiar with this product, whole blood was used from the outset of this programme for logistical reasons. Whole blood is simpler to process and MSF was providing care for large numbers of trauma patients, for whom whole blood was required. MSF did not initially diversify the type of blood product provided, as this would have increased the workload in the laboratory and limited the overall supply of donated blood that could be provided across several services. Given that even supply of whole blood was insufficient at points, this was a practical solution that maximised the provision of life-saving transfusions in a patient population with mixed clinical indications. As a high frequency of transfusion reactions were reported among the thalassaemia cohort, the use of PRC units was implemented in 2019. When supporting those with thalassaemia in conflict settings it is vital to consider the additional transfusion needs of trauma patients, who are likely to be prioritised for receiving blood products in periods of limited supply. Anticipating and planning of sufficient quantities of blood products to meet all the medical needs in the catchment area helps to ensure that those with thalassaemia have continued access. Staff recommend that stakeholders to include in blood supply strategies are local and regional health authorities, relevant armed groups, local community groups and donors, relatives, and patients.

MSF anonymously tested donated blood for blood-borne viruses (BBV) throughout the programme. Ethical challenges arose from poor access to confirmatory testing and treatment of BBV in NES, mandatory reporting to health authorities, and concerns about a lack of confidentiality, stigma, and no formally established processes for pre-test counselling and disclosing results. These factors hindered the implementation of identifiable testing and disclosure to all potential donors who consented to receive their results. Despite the lack of available treatment, MSF pursued testing and consented disclosure of results to donors and thalassaemia patients and provided counselling on how to live with BBV. MSF overcame ethical challenges by training mental health workers in pre-test counselling, informed consent, and disclosure of results to donors and thalassaemia patients who asked to be informed of their results. MSF explored referral options for those with HIV to national programmes within Syria and the provision of care for viral hepatitis within MSF facilities. However, these additional elements were not fully realised before closure of the programmes. Where safe blood transfusions have been disrupted due to health system collapse, early consideration for procedures on BBV counselling, consent, disclosure, confidentiality, and treatment will support comprehensive thalassaemia care and ensure informed and confidential care for blood donors.

#### Iron chelation therapy

MSF provided three ICT medications at different points in the programming: subcutaneous deferoxamine (DFO), oral deferiprone (DFP), oral deferasirox (DFX). All are known to reduce systemic, hepatic, and myocardial iron overload from repeated blood transfusions and are associated with increased life-expectancy of patients with thalassaemia [4]. Prior to the conflict, DFO was widely available in Syria and MSF used this iron chelator from March 2018. However, ensuring safe subcutaneous administration was difficult for many patients in precarious living conditions with poor access to running water and inadequate hygiene conditions. Oral DFX was viewed by clinical staff as easier to administer and has a good safety and efficacy profile [19], but the cost was unsustainable considering the organisation’s wider operational priorities. Given the cost barriers of DFX and administration barriers related to DFO, MSF worked with external thalassaemia experts to adapt the ICT protocol and implement oral DFP as first line treatment and in combination therapy in November 2018. The aim was to phase out use of DFX and switch the entire cohort to either DFP, DFO or a combination of both, depending on the clinical condition of the patient. Implementing the DFO and DFP ICT protocol was a financially viable option for MSF and considered acceptable in a context with high needs and no consistent access to alternative ICT.

ICT adherence counselling was a core component of MSF’s thalassaemia care. Despite a lack of trained mental health staff available in NES at the time, MSF hired and trained community mental health workers and made initiating DFP conditional on patients, and their caregiver when applicable, attending adherence counselling. Every newly enrolled individual was seen by a mental health team member. However, further specialist staff could have better met the need for developing and implementing individualised person- and family-centred care plans.

The chief safety concern of neutropenia in the first six weeks of DFP treatment was monitored using blood films, although weekly testing was problematic for patients in NES who did not live close to a laboratory, and this was one of the biggest barriers to commencing DFP treatment. When providing DFP in a complex setting with access issues, it is important to establish follow up laboratory testing in a patient accessible location. Considering the risk that neutropenia could develop between weekly testing, patients and/or caregivers were educated on the risk of neutropenia, the signs and symptoms of infection, and instructed to stop DFP immediately and seek medical care from the nearest facility if patients developed a fever. Patient-held treatment cards explained their thalassaemia diagnosis, that they were on DFP, and gave instructions to any treating physician to contact the MSF thalassaemia physician if they were presented with this card.

Although some clinical staff supported the use of DFP, others were concerned about DFP being new for the population in this context and the risk of neutropenia. MSF delayed the switch to DFP in order to sensitise health authorities, staff and patients to the new protocol and requirement for frequent follow-up. Thalassaemia department staff were trained, candidates suitable for DFP were given information about the risks and benefits, health authorities were briefed, clinical preparations (such as follow up charts for blood counts) were made, and the laboratory prepared by implementing corrected white blood count, differential, and morphology for blood films. To maximise patient and family buy-in, counsellors and community health workers gave information about DFP to families, informing them that all patients will switch as there will be no further supply of DFX by MSF. Some staff and patients remained hesitant, although this improved over time.

By April 2019, those on DFP were reportedly documented as showing marked improvement in ferritin levels compared to those receiving DFO and DFX, although this cannot be confirmed by the available clinical data.

#### Continuity of care and patient access

In 2018, the management threshold for all MSF programmes in NES was reached after scaling up a conflict related response to support massive numbers of wounded patients from Raqqa [20]. Despite ongoing medical humanitarian needs in Menbij, MSF closed the project in April 2018 due to competing medical priorities in the region. According to local community reports to staff as well as assessments done by staff, MSF’s thalassaemia programme in Tal Abyad remained the only service providing comprehensive thalassaemia care, including newly initiated ICT, in non-Syrian government-controlled areas in NES at that time. However, periods of heightened conflict in the region also affected the provision of thalassaemia care in Tal Abyad. Greater security risks led to a reduction in staffing and the remaining staff were prioritised to support higher acuity departments such as trauma and maternity.

A large proportion of the patients attending the Tal Abyad clinic resided in Raqqa city and, although implementing a comprehensive thalassaemia programme in that location was not feasible, MSF arranged bus transport between the two locations to facilitate access. However, patients were not always able to take pre-booked transport so their arrivals did not always coordinate well with their clinical needs, and clinics were sometimes overcrowded and overwhelmed when many patients arrived together by bus. Transport timetables coordinated with clinic appointments that were co-designed with communities may have better met the needs of patients and solved clinic overcrowding and delays in follow-up. MSF also expanded provision of laboratory services to Raqqa with clear referral criteria for follow-up in the main clinic in Tal Abyad, which reduced the frequency of long-distance travel for patients. Service flexibility to accommodate patients and care givers as they are available is vital in conflict settings where people often experience significant competing priorities that hinder frequent attendance and/or predefined appointment dates.

When blood bank capacity expanded in Raqqa city through other health actors, and in recognition that patients were accessing transfusions outside of the MSF Tal Abyad programme, patient-held thalassaemia records were introduced to capture the clinical history, test results, and treatments regardless of which facility they attended. This enabled some continuity of care; at minimum it facilitated patient information to be available to any treating physician.

The Microsoft Excel-based thalassaemia data tool did not collect clear information on all patient variables (e.g., ferritin levels and clinical outcomes) and data entry was burdensome and often incomplete. This was a barrier to monitoring individual patients’ clinical progress on a long-term basis. This also prevented the analysis of the cohort as a whole, which challenged the planning for medical supply and human resources needs, as well as evaluation of the programme.

Thalassaemia clinic data show higher attendance of patients residing in sub-districts served by MSF’s non-thalassaemia related medical programmes, and those areas were thought to have created an access point for referral to the MSF thalassaemia clinics. Although the total population of people living with thalassaemia in each region or sub-district was not available to determine if this is proportionate, it indicates that there may have been lower awareness of care options and therefore lower uptake in areas not served by any MSF programmes.

MSF’s sudden loss of access to Tal Abyad in October 2019 led to a loss of all ICT medications. Despite frequent shifts in frontlines over the period of the conflict in NES, ICT stock was held in one location. Division of stock between multiple locations in this context may have enabled ongoing care provision should a portion of the supplies have become inaccessible due to insecurity.

#### Supportive care and quality of life

In addition to adherence counselling, wider psychosocial support and care was a priority for thalassaemia programming. Its implementation was challenged by a lack of trained staff, a lack of supervision, high mental health needs in other patient groups creating a competition for limited resources, and stigma. There were high rates of defaulters from mental health services reported by staff, although data captured on this was lacking which further challenged follow up. Some of these challenges were overcome and many patients and families took part in counselling on thalassaemia and its complications, group sessions, play sessions, educational activities, school, and ‘Thalassaemia Day’ celebrations. Informal networks developed between the families of people living with thalassaemia and with staff which supported continuity of care and sharing of information. Caregivers of children living with thalassaemia were encouraged to facilitate social activity groups and educational materials were developed and shared.

ICT was reported to improve the clinical condition of patients, although quality of life indicator data demonstrating this were not captured. With the initiation of ICT, alongside mental health care and support, staff reported seeing positive psychological effects in the thalassaemia cohort and their families [17]. The sudden closure of activities resulted in lost contact between MSF staff and patients and their families, so quality of life changes, including the impact of interrupted provision of thalassaemia care and ICT cannot be retrospectively explored. The disruption to a life-sustaining service may have had a negative impact on families and patients, some of whom endured the loss of access to healthcare more than once. However, it can be argued that the provision of any thalassaemia care, even if limited in time, is worthwhile.

#### Collaboration with local staff

Those who worked on thalassaemia care were largely local Syrian staff. Although prior to the war there was a high level of thalassaemia expertise in Syria, most local staff in MSF project locations at that time did not have previous experience with thalassemia care. Internationally mobile and headquarters staff supported the activities with training and guidelines. A local expert in thalassaemia also shared knowledge with internationally mobile MSF staff.

## Discussion

Several contextual and operational factors strengthened or challenged MSF’s thalassaemia programmes in NES including population displacement, challenges in access, the destruction of medical facilities, and periods of insecurity associated with the evolving conflict [2, 17, 21]. As provision of thalassaemia care within an MSF project was novel, MSF staff did not have the necessary knowledge or experience to roll-out a highly efficient approach from the outset. MSF clinicians with experience in the implementation of complex care programmes in low resource contexts worked with local Syrian healthcare colleagues and external experts from outside Syria to evolve the programme design over time adapting to learning about this patient population and context [1].

In this protracted conflict setting, MSF implemented key elements of thalassaemia programming with core elements of what were the then international recommendations, usually reserved for well-resourced services in stable contexts [5]. This includes the provision of day care with access to inpatient facilities. In addition, evidence-based protocols for the provision of comprehensive and holistic care were modified to the setting. For example, although DFP is recommended as second-line ICT in international guidelines and high-income settings such as Europe, the United States, and the United Kingdom [22–25], it is appropriate to select treatment based on an individual assessment of toxicity, tolerability, and adherence [26]. MSF’s practice reflected the reality of the patients’ environment with consideration for social factors and clinical indicators which underpinned toxicity, tolerability, and adherence and determine success of ICT. MSF implemented regimens of ICT and monitoring that were more affordable and reflected the realities of patients’ living conditions and limited clinic access. Price negotiation to make medications more affordable for humanitarian organisations has the potential for creating widened access for thalassaemia patients in conflict settings.

MSF also facilitated more equitable access via provision of patient transport, expansion of laboratory services closer to the origin of people in the cohort, and patient held medical records facilitated continuity of care between clinics. Given the importance of regular access to thalassaemia care for transfusions and ICT follow up care, and the disruptive impact of conflict and insecurity on staff presence in clinics and patients’ ability to access fixed clinic locations, providers should consider the allocation of exclusive resources, including staffing and satellite care facilities, for thalassaemia programmes in order to maximise access and continuity of care. Where regional healthcare systems are disrupted, awareness and sensitisation activities in communities which are likely to have similar rates of genetic conditions may generate additional demand and meet needs appropriately. Where a lack of personal finances and insecurity limits transportation, organisations should decentralise care using knowledge of potential service providers and engage medical staff and community members to determine the appropriate model to create access. This could range from transport provision for patients or their samples, to bringing laboratory services closer to patients with a feedback mechanism to a main clinic location, to provision of a full package of care in multiple locations.

MSF’s thalassaemia programme differs from international recommendations in that blood bank and laboratory services were provided ‘in house’ negating the need for ‘close collaboration’ across services, although challenges in supply and efficiency affected care. The importance of safe blood transfusions supported by good quality blood bank and laboratory services is clear. In 2018, the thalassaemia patient cohort had a hepatitis C virus (HCV) positivity rate of 28% (n = 44/155) [17]. This is significantly higher than estimated national rates of 2.85% for HCV infection for the same year and reflects the lack of access to safe care during the conflict, which forced families to take risks, including unsafe blood transfusions [1, 27].

Patient support activities were provided and the creation of informal networks were facilitated which partly meets the recommendation to collaborate with patient support groups. MSF also worked with and advocated to health authorities for service developments, such as the implementation of ICT, and patients’ rights to access care [1, 17].

Finally, there was no formal participation in research, however this case study aims to highlight gaps in evidence on implementation of thalassaemia care in humanitarian settings. Ensuring good data collection tools will not only support research but improve patient monitoring and programme evaluation. When providing highly specialised physical, social, and mental health care for a multisystem condition, understanding the impact on patients and their families is important for evaluating the success of the programme. The incorporation of quality-of-life indicators into data collection will facilitate this. Possible tools for measuring quality of life include the TranQol (Transfusion-dependent Quality of Life Questionnaire) measure of quality of life for patients with thalassaemia [28]. This may be suitable for intermittent measurements throughout treatment. However, some topics may not be relevant to conflict settings so this tool should be adapted to the setting and piloted.

### Limitations

A key limitation of this case study is the lack of Syrian authorship. Inclusion of Syrian colleagues’, patients’, and families’ experiences of thalassaemia care, MSF’s approach, and understanding of the health system and context in non-Syrian government-controlled areas of NES between April 2017 and October 2019 would enrich the findings, address gaps in knowledge, correct any errors in understanding, and ensure a more realistic interpretation of results. Sudden loss of access to the community and colleagues prevented their inclusion, so the work is restricted to the understanding and perspectives of authors with more limited experiences of NES. Further, staff were asked for their views and to confirm details about the programme between one and a half and four years after their period of engagement with thalassaemia programming in NES. The time lag may affect the accuracy of their recall.

The period of the thalassaemia programme reviewed in this article preceded the use of a central storage of programme information in MSF, so it is possible that some documentation was not included in the analysis. Although some findings from previous clinical analyses are included [12, 17], new analysis of routine thalassaemia patient data collected between 2017 and 2019 was limited to a cohort description. Challenges in data collection when working in conflict zones are noted to be a barrier to developing evidence in expert interviews in the literature [15]. MSF’s programme also struggled to maintain complete high-quality data on risk factors, clinical and quality of life outcomes are not available. Therefore, a formal evaluation of whether thalassaemia programming in NES met its expected clinical objectives and/or the extent to which changes in individual outcomes can be attributed to the programme was out of scope for this case study.

## Conclusions

MSF provided outpatient thalassaemia programmes in NES from April 2017 to October 2019 in a complex conflict setting challenged by population displacement, the destruction of medical facilities, reduction in healthcare staff, a disrupted healthcare system, and periods of high insecurity. These programmes were novel for MSF and operational learning was taking place alongside implementation. Here we have reflected on lessons identified in delivering thalassaemia investigations, blood transfusion, and iron chelation therapy, continuity of care and patient access, and psychosocial support and quality of life. We have made key recommendations for programming based on lessons identified. Although programming was not “routine”, MSF has demonstrated that life-sustaining thalassaemia care can be provided in complex conflict settings and international non-governmental organisations can consider this care feasible in similar contexts.

## Data Availability

MSF has a managed access system for data sharing that respects MSF’s legal and ethical obligations to its patients to collect, manage and protect their data responsibly. Ethical risks include, but are not limited to, the nature of MSF operations and target populations being such that data collected are often highly sensitive. Data are available on request in accordance with MSF's data sharing policy (available at: http://fieldresearch.msf.org/msf/handle/10144/306501). Requests for access to data should be made to data.sharing@msf.org.

## Abbreviations

BBV: Bloodborne viruses
DFO: Deferoxamine
DFP: Deferiprone
DFX: Deferasirox
HCV: Hepatitis C virus
HIV: Human immunodeficiency virus
ICT: Iron chelation therapy
MSF: Médecins Sans Frontières
NES: Northeast Syria
PRC: Packed red cells
SDF: Syrian Democratic Forces

## References

[1] MSF. Syria: treating patients with chronic conditions in a war context | MSF. 2018. https://www.msf.org/syria-treating-patients-chronic-conditions-war-context. Accessed 10 Aug 2020.

[2] MSF. A decade of war in Syria: 10 years of increasing humanitarian needs. 2021. https://www.msf.org/decade-war-syria. Accessed 4 Mar 2021.

[3] Hamamy HA, Al-Allawi NAS (2013). Epidemiological profile of common haemoglobinopathies in Arab countries. J Community Genet.

[4] Taher AT, Musallam KM, Cappellini MD (2021). β-Thalassemias. N Engl J Med.

[5] Angastiniotis M, Eleftheriou A, Cappellini MD, Taher A, Cappellini MD, Cohen A, Porter J, Taher A, Viprakasit V (2014). Organisation and programming of thalassaemia care. Guidelines for the management of transfusion dependent thalassaemia (TDT).

[6] Healy S, Aneja U, DuBois M, Harvey P, Poole L. Working with local actors: MSF’s approach. 2019. https://odihpn.org/publication/working-with-local-actors-msf/. Accessed 29 Oct 2022.

[7] United Nations Office for the Coordination of Humanitarian Affairs (OCHA). Syria Crisis: Ar-Raqqa—Situation Update No. 1 (as of 31 January 2017). 2017. https://reliefweb.int/report/syrian-arab-republic/syrian-arab-republic-ar-raqqa-situation-report-no-1-31-january-2017-enar. Accessed 18 Nov 2022.

[8] United Nations Office for the Coordination of Humanitarian Affairs (OCHA). Syria Crisis: Menbij and Ar-Raqqa—Situation Report No. 3. 2017. https://reliefweb.int/report/syrian-arab-republic/syria-crisis-menbij-and-ar-raqqa-situation-report-no-3-8-april-2017. Accessed 7 Nov 2022.

[9] United Nations Office for the Coordination of Humanitarian Affairs (OCHA). Syria Crisis: Northeast Syria—Situation Report No. 27. 2018. https://reliefweb.int/report/syrian-arab-republic/syria-crisis-northeast-syria-situation-report-no-27-15-july-2018-31. Accessed 7 Nov 2022.

[10] United Nations Office for the Coordination of Humanitarian Affairs (OCHA). Humanitarian Update—Syrian Arab Republic: Issue 6 [Internet]. 2019. https://reliefweb.int/report/syrian-arab-republic/humanitarian-update-syrian-arab-republic-issue-06-14-november-2019. Accessed 14 Nov 2022.

[11] Vernier L, Cramond V, Hoetjes M, Lenglet A, Hoare T, Malaeb R (2019). High levels of mortality, exposure to violence and psychological distress experienced by the internally displaced population of Ein Issa camp prior to and during their displacement in Northeast Syria, November 2017. Confl Health.

[12] Meiqari L, Hoetjes M, Baxter L, Lenglet A (2018). Impact of war on child health in northern Syria: the experience of Médecins Sans Frontières. Eur J Pediatr..

[13] MSF. Northeast Syria: MSF evacuates staff due to extreme volatility. 2019. https://www.msf.org/northeast-syria-msf-forced-evacuate-staff-due-extreme-volatility-region. Accessed 16 Apr 2021.

[14] MSF. Changing young lives in Lebanon: Thalassemia in the shadow of COVID-19. 2020. https://msf.org.uk/article/changing-young-lives-lebanon-thalassemia-shadow-covid-19. Accessed 21 Apr 2021.

[15] Blanchet K, Roberts B, Sistenich V, Ramesh A, Frison S, Warren E, et al. An evidence review of research on health interventions in humanitarian crises Principal Investigators. 2015.

[16] Bolt M, Schoneboom B (2010). Operative splenectomy for treatment of homozygous thalassemia major in Afghan children at a US military hospital. AANA J.

[17] Foley D, Aoki Y, Roggeveen H, Carrion-Martin AI, Mullahzada AW, Hoetjes M. The forgotten frontline… improving thalassaemia outcomes with iron chelation therapy in a conflict setting. MSF Paediatric Days. 2019. https://www.researchgate.net/publication/332801399_The_Forgotten_Frontline_Improving_Thalassaemia_Outcomes_with_Iron_Chelation_Therapy_in_a_Conflict_Setting. Accessed 9 Mar 2021.

[18] Trompeter S, Cohen A, Porter J, Cappellini MD, Cohen A, Porter J, Taher A, Viprakasit V (2014). Blood transfusion. Guidelines for the management of transfusion dependent thalassaemia (TDT).

[19] Algren DA. Review of oral iron chelators (Deferiprone and Deferasirox) for the treatment of iron overload in pediatric patients. 2010.

[20] Okeeffe J, Vernier L, Cramond V, Majeed S, Carrion Martin AI, Hoetjes M (2019). The blast wounded of Raqqa, Syria: observational results from an MSF-supported district hospital. Confl Health.

[21] Devi S (2021). Health in Syria: a decade of conflict. Lancet.

[22] European Medicines Agency. Ferriprox. 2021. https://www.ema.europa.eu/en/documents/product-information/ferriprox-epar-product-information_en.pdf. Accessed 19 Apr 2021.

[23] US Food and Drug Administration. FERRIPROX^®^ (deferiprone) tablets, for oral use. 2011. https://www.accessdata.fda.gov/drugsatfda_docs/label/2011/021825lbl.pdf. Accessed 19 Apr 2021.

[24] National Institute for Health and Care Excellence. DEFERIPRONE | Drug | BNF content. 2021. https://bnf.nice.org.uk/drug/deferiprone.html. Accessed 19 Apr 2021.

[25] Porter J, Viprakasit V, Kattamis A, Cappellini MD, Cohen A, Porter J, Taher A, Viprakasit V (2014). Iron overload and chelation. Guidelines for the management of transfusion dependent thalassaemia.

[26] Specialised Commissioning Team NHS England. Clinical Commissioning Policy: Treatment of iron overload for transfused and non transfused patients with chronic inherited anaemias. 2016. https://www.england.nhs.uk/wp-content/uploads/2018/07/Treatment-of-iron-overload-for-transfused-and-non-transfused-patients-with-chronic-inherited-anaemias.pdf. Accessed 19 Apr 2021.

[27] Coalition for Global Hepatitis Elimination. Syrian Arab Republic. 2021. https://www.globalhep.org/country-progress/syrian-arab-republic. Accessed 28 Apr 2021.

[28] Klaassen RJ, Barrowman N, Merelles-Pulcini M, Vichinsky EP, Sweeters N, Kirby-Allen M (2014). Validation and reliability of a disease-specific quality of life measure (the TranQol) in adults and children with thalassaemia major. Br J Haematol.

